# Low Absolute Eosinophil Count Predicts In-Hospital Mortality in Cirrhosis With Systemic Inflammatory Response Syndrome

**DOI:** 10.7759/cureus.12643

**Published:** 2021-01-11

**Authors:** Varsha Wilson, Kunnothara Kantan Velayudhan, Harshavardhan Rao, Sheejamol Velickakathu Sukumaran

**Affiliations:** 1 Internal Medicine, Amrita Institute of Medical Sciences and Research, Kochi, IND; 2 Gastroenterology, Amrita Institute of Medical Sciences and Research, Kochi, IND; 3 Biostatistics, Amrita Institute of Medical Sciences and Research, Kochi, IND

**Keywords:** absolute eosinophil count, cirrhosis, chronic liver disease, mortality, systemic inflammatory response syndrome

## Abstract

Introduction

Chronic liver disease (CLD) or Cirrhosis is one of the most common causes of morbidity as well as mortality. Child-Turcotte-Pugh (CTP) score and the model for end-stage liver disease (MELD) are useful to assess the long-term prognosis of a patient with CLD. When a patient with CLD is admitted with an acute illness leading to systemic inflammatory response syndrome (SIRS), these scores may not be reliable to predict the short-term prognosis and survival. Absolute eosinophils count (AEC) allows the rapid identification of patients at increased risk for sepsis-related mortality in patients.

Methods

This was a cross-sectional study conducted among patients in a tertiary care hospital in South India during a period of one and a half years between October 2018 and April 2020. Cirrhotic patients with SIRS aged between 16 years and 80 years were included in the study. AEC was measured as a part of automated complete blood counts. Patient demographics, lab parameters, and outcomes in terms of mortality were studied. Continuous variables were expressed as mean ± SD/median and categorical variables were expressed in frequency. Receiver operating characteristic (ROC) curve analysis was used to find an ideal cutoff for AEC in predicting hospital mortality. Multi-variate Cox regression analysis was performed to find predictors of mortality.

Results

A total of 100 patients who fit the pre-determined criteria for cirrhosis with SIRS were enrolled in the study. Sixteen (16%) patients died at the end of the study while 84 (84%) were alive. Using a ROC curve, the area under the curve (AUC) was 0.716 with 95% CI of AUC (0.564-0.867), the p-value was found to 0.006, a cut-off of eosinophil count of 198.5 cells/uL was found to be the cut-off for the prediction of in-hospital mortality in this subset of patients with cirrhosis and sepsis with SIRS, with a sensitivity of 75% and specificity of 38.1%. In a multi-variate Cox regression analysis, only age (hazard ratio {HR}: 1.175, 95%CI, 1.084 to 1.275, p<0.001) , CRP (HR : 1.008, 95%CI, 1.00 to 1.015, p=0.042) values, total leukocyte counts (TLC) (HR: 1.226, 95%CI, 1.116 to 1.346, p<0.001) and AEC (HR: 0.993, 95%CI, 0.987 to 0.999, p=0.016) were found to be statistically significant independent predictors of mortality.

Conclusions

The presence of eosinopenia may be considered as an in-expensive warning biomarker for poorer clinical outcomes in the form of in-hospital mortality in hospitalized cirrhotic patients. Other biomarkers such as CRP and TLC could also play a role both independently and in conjunction with AEC to predict outcomes and mortality in cirrhotic patients with sepsis and SIRS.

## Introduction

Chronic liver disease (CLD) or cirrhosis is one of the most common causes of morbidity as well as mortality worldwide, but especially so in the developing world [1]. The etiology behind CLD includes, but is not limited to alcoholic liver disease, non-alcoholic fatty liver disease (NAFLD or NASH), chronic viral hepatitis, genetic causes like alpha-1 antitrypsin deficiency, hereditary hemochromatosis, Wilson’s disease, autoimmune causes such as primary biliary cirrhosis (PBC), primary sclerosing cholangitis (PSC), autoimmune hepatitis (AIH), and other causes such as drugs (amiodarone, isoniazid, methotrexate, phenytoin, nitrofurantoin), vascular (Budd-Chiari syndrome), and idiopathic/cryptogenic CLD [1].

CLD involves a continuous process of hepatic fibrosis, alteration of the liver tissue architecture, and formation of regeneration nodules [1]. The systemic manifestations and complications range from variceal bleeding, ascites and spontaneous bacterial peritonitis (SBP), hepatic encephalopathy, hepatorenal syndrome, hepatopulmonary syndrome, hepatocellular carcinoma (HCC) and can also involve the cardiac and musculoskeletal systems [1]. Sepsis and systemic inflammatory response syndrome (SIRS) is one of the most common complications in CLD; whatever be the etiology. There are several severity scores for the assessment of the prognosis of a patient of CLD, such as the Child-Turcotte-Pugh (CTP) score and the model for end-stage liver disease (MELD).

Numerous studies have found that eosinopenia is an accurate marker of bloodstream infections, especially in critically ill patients [2-4]. Absolute eosinophils count (AEC) allows for the rapid identification of patients at increased risk for sepsis-related mortality in patients [5]. Because the eosinophil count is routinely done as part of the complete blood count in almost all patients, and especially in septic patients, it does not entail an extra effort or cost to the patient. Hence, the AEC has been reported to have the necessary sensitivity and specificity coupled with the ease and the benefit of being cost-effective, which is not seen with other markers for sepsis

## Materials and methods

This was a cross-sectional study done among cirrhotic patients with features of SIRS during a period of one and a half years (October 2018 to April 2020). Cirrhotic patients with SIRS aged between 16 years and 80 years were included in the study.

Selection and description of patients

Cirrhotic patients who fulfilled the criteria for SIRS were included in the study. Patients who died within 24 hours of admission, who had a concomitant malignancy, who had a severe cardio-pulmonary illness, who had documented allergic disorders (e.g., asthma, hay fever, allergic skin diseases such as pemphigus and dermatitis herpetiformis), or with certain autoimmune disorders (e.g., systemic lupus erythematosus, vasculitis), or on steroids or other immunosuppressive therapy were excluded from the study. Cirrhotic patients who had bacteremia or suspected sepsis without SIRS were not included in the study. Based on the sensitivity of AEC (78.5%) in predicting hospital mortality among Cirrhotic patients with SIRS, observed in an earlier publication by Kotecha et al., and with 20% allowable error and 95% confidence, the minimum sample size came to 16 [6]. Finally, a sample size of 100 patients was included.

Objectives and patient definitions

Cirrhosis was diagnosed based on clinical, radiological, or histological criteria. SIRS was defined in accordance with the American College of Chest Physicians/Society of Critical Care criteria by the presence of two or more of the following parameters: body temperature >38 C or <36 C; heart rate >90beats/min; respiratory rate >20/min or PaCO2 <32 mmHg; white blood cell count >12,000 or <4000 cells/cumm, or >10% immature forms. Sepsis was defined as SIRS associated with the presence of an infectious process [6]. The primary objective was to report the diagnostic accuracy of AEC at the time of admission in predicting in-hospital mortality among cirrhotic patients with SIRS. AEC was measured as a part of automated CBC. According to the inclusion criteria, patient demography in the form of age, gender, comorbid illnesses, CTP and MELD scores, and other investigations pertaining to sepsis were captured. CTP and MELD scores were determined using the worst values within the initial 24 hours of admission to assess the severity of underlying CLD. The patients were followed up until discharge or mortality. 

Statistical methods

Statistical analysis was done using the IBM Statistical Package for the Social Sciences (SPSS) Statistics 20 Windows (SPSS Inc., Chicago, USA). The results are given in mean ± SD/median (interquartile range {IQR}) for all the continuous variables and frequency (percentage) for categorical variables. Receiver operating characteristic (ROC) curve analysis will be used to find an ideal cutoff for AEC in predicting hospital mortality. To test the statistical significance of the association of AEC with Hospital mortality, a chi-square test was used. Diagnostic measures such as sensitivity, specificity, positive predictive value (PPV), negative predictive value (NPV), and accuracy were calculated. To test the statistical significant difference in the average parameters between two groups, the independent sample t-test was used in the case of normality and the Mann-Whitney U test in the case of non-normality. Univariate Cox regression and stepwise multi-variate Cox regression analysis were used to predicting significant independent factors for mortality. P-value of <0.05 was considered statistically significant. All tests of statistical significance were two-tailed

## Results

A total of 100 patients who fit the pre-determined criteria for cirrhosis with sepsis and SIRS were enrolled in the study. Sixteen (16%) patients died at the end of the study while 84 (84%) were alive.

There were 79 (79%) males and 21 (21%) females enrolled in the study. The ages in this study ranged between 31 years and 77 years of age. The mean age of the patient was 59.17±10.17 years. A total of 64 patients had an alcoholic etiology for cirrhosis, 13 patients had NASH, nine patients had autoimmune etiology, seven patients had a cryptogenic etiology, six patients had viral hepatitis, and one patient had Wilson’s disease.

A univariate Cox regression analysis showed age (hazard ratio {HR}: 1.141, 95%CI, 1.060 to 1.228, p<0.001), TLC (HR: 1.126, 95%CI, 1.067 to 1.189, p<0.001), platelet counts (HR: 1.012, 95%CI, 1.003 to 1.021, p=0.008), AEC (HR: 0.992, 95% CI, 0.986 to 0.998, p=0.012), CRP (HR: 1.015, 95%CI, 1.010 to 1.020), serum albumin (HR: 0.192, 95% CI, 0.074 to 0.502, p=0.001), and serum creatinine (HR: 1.401, 95%CI, 1.140 to 1.723, p=0.001) were found to be statistically significant predictors for mortality. Performing a stepwise multi-variate Cox regression analysis of all the biochemical parameters studied, only age (HR: 1.175, 95%CI, 1.084 to 1.275, p<0.001), CRP (HR: 1.008, 95%CI, 1.00 to 1.015, p=0.042) values, TLC (HR: 1.226, 95%CI, 1.116 to 1.346, p<0.001) and AEC (HR: 0.993, 95%CI, 0.987 to 0.999, p=0.016) were found to be statistically significant independent predictors for mortality (Tables 1, 2). The comparison between the various studied parameters in the mortality and alive groups are summarized in Table 3. 

**Table 1:** Univariate Cox regression analysis CRP: C-reactive protein, ALT: alanine aminotransferase, AST: aspartate aminotransferase, INR: international normalized ratio, CTP: Child-Turcotte-Pugh, MELD: model for end-stage liver disease

Parameter	Hazard ratio	95% Confidence interval lower limit	95% Confidence interval upper limit	P-value
Age	1.141	1.060	1.228	<0.001
Gender	1.901	0.432	8.366	0.395
Hemoglobin	0.918	0.725	1.161	0.475
Total leukocyte counts	1.126	1.067	1.189	<0.001
Platelet counts	1.012	1.003	1.021	0.008
Absolute eosinophil counts	0.992	0.986	0.998	0.012
CRP	1.015	1.010	1.020	<0.001
Total bilirubin	1.028	0.926	1.141	0.607
ALT	0.995	0.971	1.019	0.659
AST	0.983	0.959	1.008	0.176
Serum protein	0.801	0.519	1.236	0.316
Serum albumin	0.192	0.074	0.502	0.001
INR	0.869	0.374	2.016	0.743
Serum creatinine	1.401	1.140	1.723	0.001
Serum sodium	1.022	0.940	1.112	0.607
CTP score	1.631	0.612	4.345	0.328
MELD score	0.989	0.925	1.056	0.733

**Table 2:** Multi-variate Cox regression analysis CRP: C-reactive protein

Parameter	Hazard ratio	Confidence interval (95%) lower limit	Confidence interval (95%) upper limit	P-value
Age	1.175	1.084	1.275	<0.001
CRP	1.008	1.000	1.015	0.042
Total leukocyte counts	1.226	1.116	1.346	<0.001
Absolute eosinophil counts	0.993	0.987	0.999	0.016

**Table 3:** Comparison between the study parameters in alive and mortality groups CRP: C-reactive protein, ALT: alanine aminotransferase, AST: aspartate aminotransferase, INR: international normalized ratio, CTP: Child-Turcotte-Pugh, MELD: model for end-stage liver disease. ^1^Expressed in terms of number of samples. ^2^Expressed in terms of mode score.

Parameter	Alive (n=84)	Mortality (n=16)
Age (years)	57.6±9.9	67.3±7.9
Male gender^1^	14 (87.5%)	65 (77.3%)
Hemoglobin (g/dL)	9.8±2.2	9.4±1.5
Total leukocyte counts (cells x 10^9^/L)	5.7±0.4	12.4±1.2
Platelet counts (cells x 10^9^/L)	90.9±4.8	124.8±14.4
Absolute eosinophil counts (cells/uL)	213.8±17.8	108±25.2
C-reactive protein (mg/dL)	13.9±1.7	88.7±18.8
Total bilirubin (mg/dL)	3.4±0.4	3.8±1.7
ALT (U/L)	31.3±3.3	27.8±6.4
AST (U/L)	50.5±3.8	37.8±4.9
Serum protein (g/dL)	6.7±1	6.4±0.8
Serum albumin (g/dL)	3±0.6	2.4±0.2
INR	1.6±0.6	1.6±0.3
Serum creatinine (mg/dL)	1.3±0.1	2.4±0.5
Serum sodium (mEq/L)	134.1±6.3	134.8±4.5
CTP score^2^	B	C
MELD score	17.6±7.5	16.9±8.3

**Figure 1:**
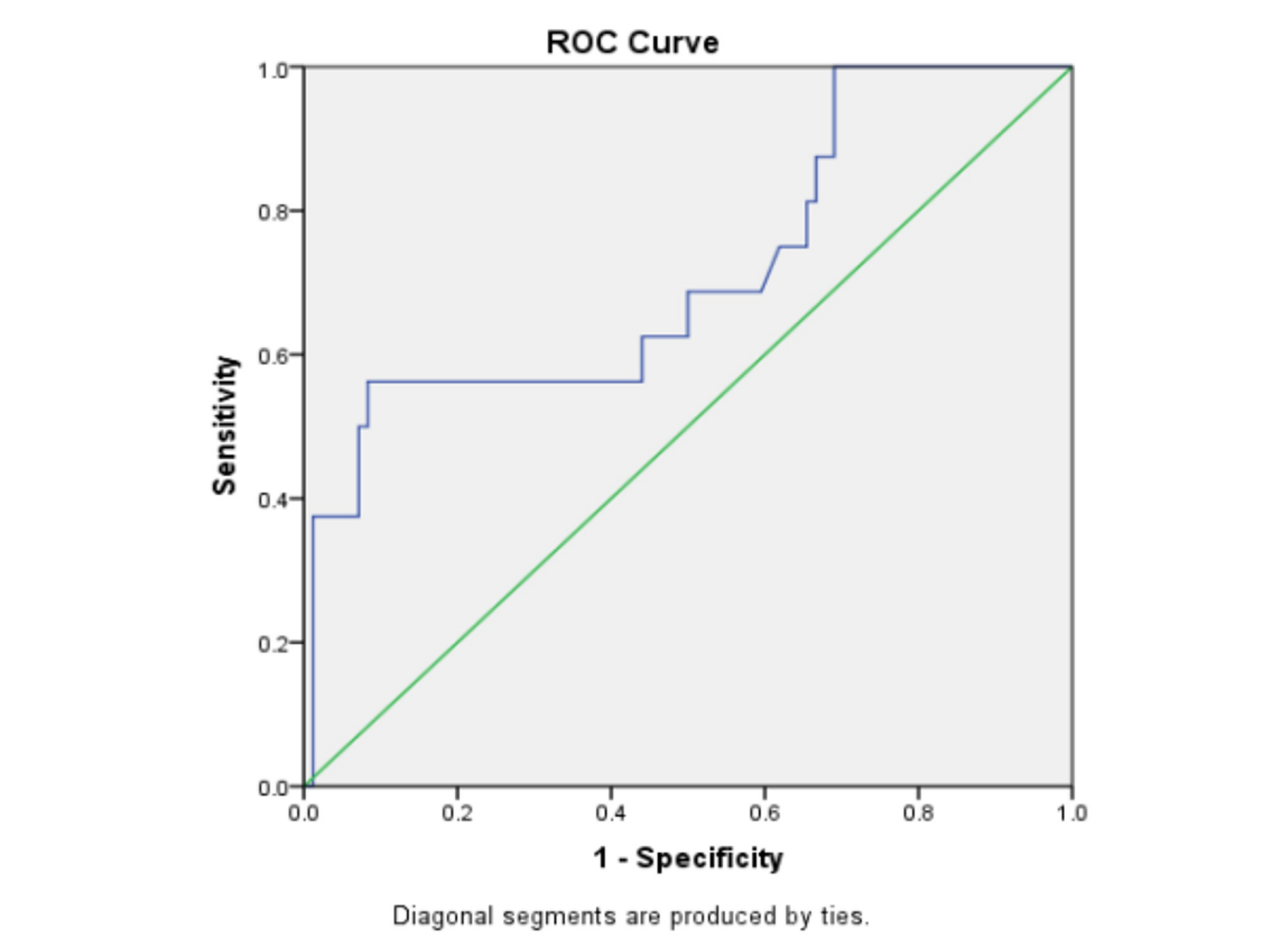
ROC curve of the absolute eosinophil count ROC: receiver operating characteristic

Using a receiver operating characteristic curve, the area under the curve (AUC) was 0.716 with 95%CI of AUC (0.564-0.867), the p-value was found to 0.006, a cut-off of eosinophil count of 198.5 cells/uL was found to be the cut-off for the prediction of in-hospital mortality in this subset of patients with cirrhosis and sepsis with SIRS, with a sensitivity of 75% and specificity of 38.1% (Figure 1). A total of 64 (64%) patients had an AEC below 198.5 cells/uL (cut-off value) while 36 (36%) patients had an AEC above it. In the mortality group, 12 patients (75%) patients had an AEC below the cut-off value, while four patients (25%) did not. In the alive group, 52 patients (62%) had an AEC below the cut-off value while 32 patients (38%) did not (Tables 2, 3). The sensitivity, specificity, positive predictive value, negative predictive value, and diagnostic accuracy of AEC cut-off value of 198.5 for predicting mortality were 75%, 38.1%, 18.7%, 88.9%, and 44%, respectively.

## Discussion

This study showed that in cirrhotic patients with sepsis and SIRS, the ROC curve showed that a baseline AEC value of <198.5 cells/cumm may be used to reasonably predict the in-hospital mortality with high sensitivity. The prediction of mortality by AEC was found to be independent of the other studied markers, including the MELD and CTP scores. The diagnostic accuracy of AEC was however only found to be reasonable at around 44%. Hence, in addition to well-established biomarkers like CRP and TC, along with established scoring systems such as Sequential Organ Failure Assessment (SOFA) score or Acute Physiology and Chronic Health Evaluation (APACHE) II scores, the presence of eosinopenia may be considered as an in-expensive warning biomarker for poorer clinical outcome in the form of in-hospital mortality in hospitalized cirrhotic patients.

The MELD and CTP scores seem to be more useful in the long-term prognosis of cirrhotic patients and not in acutely ill patients with CLD. Hence, AEC may be superior to scoring systems such as CTP and MELD in predicting short-term in-hospital mortality in acutely ill cirrhotic patients. However, the role of AEC in association with established scoring systems and more conventional biomarkers such as procalcitonin (PCT) may warrant further study.

Other biomarkers such as CRP, serum creatinine, total leukocyte count, platelet count could also play a role both independently and in conjunction with AEC to predict outcomes and mortality in cirrhotic patients with sepsis and SIRS. Wibrow et al. reported that the presence of eosinopenia can be used as an inexpensive marker for sepsis in hospitalized adult patients [7]. Our study also closely resembled the findings of Kotecha et al who reported that the MELD score, serum sodium, and AEC were all predictors of mortality in cirrhotic patients admitted with SIRS [6]. In addition, significant associations were found between increasing age and mortality even though no correlation was found with gender. This was in keeping with reports by Martin et al. who reported that age was associated with both the development of and adverse outcomes of sepsis [8]. Angus et al. reported that there was a steadily increased mortality associated with age, with a highly significant peak of about 40% in patients >85 years [9]. Blot et al. reported in a large cohort study that mortality rates went up with age: about 43%, 49%, and 56% in middle-aged, old, and very old patients [10].

Another finding of this study included the association between lower serum albumin levels and increased mortality. Kendall et al. reported that albumin is an independent predictor of mortality in critically ill septic patients [11]. Similar findings were also reported by Takegawa et al. who in addition to serum albumin, found that total protein could also help predict mortality in sepsis. However, we could not reproduce those results in this study [12]. Other markers of interest in predicting mortality included the total leukocyte counts, platelet counts, CRP, and serum creatinine. Each of these associations has been independently reported in published literature. Aminzadeh et al. established a relationship between increasing leukocyte counts and significantly worse outcomes in septic patients [13]. Devran et al. reported that CRP appeared to be as valuable a marker for the prediction of mortality as the SOFA score in septic patients [14]. In another study, mean CRP for mortality and survivor groups were 174 mg/L and 85.6 mg/L, respectively with an established association between higher mortality with higher CRP [14]. The relationship between higher creatinine values and platelet values with an increased mortality is very well established, and they are both components of the SOFA score to predict mortality in sepsis [15].

Even though multiple parameters showed promise individually, a multi-variate analysis and Cox regression revealed that the only four parameters with significant association to mortality were age, CRP, TLC, and AEC. Further studies with larger sample sizes may be required to establish for certain whether there exists a relationship between the other parameters, specifically AEC, and mortality in multi-variate analyses.

This study has its limitations. First, even though the sample size was calculated based on established prevalence, it could still be under-powered to assess the potential predictive ability of eosinopenia as a marker of mortality. Second, we did not collect clinical data other than age, gender, CTP, and MELD status of cirrhosis, and mortality of the study patients. Other clinical data like co-morbid illnesses, the SOFA or APACHE scores, etc may provide additional information. We did not include procalcitonin, which may be a better prognostic marker of sepsis than CRP. Finally, even though every attempt was made to avoid confounders that can influence the AEC value, there could have been other conditions such as helminthic infections which have a high endemicity in the geographical area. 

## Conclusions

The presence of eosinopenia may be considered as an in-expensive warning biomarker for poorer clinical outcomes in the form of in-hospital mortality in hospitalized cirrhotic patients. In critically ill cirrhotic patients with SIRS, a baseline AEC value < 198.5 cells/mm3 can accurately predict the in-hospital mortality with high sensitivity. Other biomarkers such as CRP, serum creatinine, total leukocyte count, platelet count could also play a role both independently and in conjunction with AEC to predict outcomes and mortality in cirrhotic patients with sepsis and SIRS.

Further studies are indicated to establish this role of AEC in the prediction of outcomes in cirrhotics with sepsis and also its relationship with other biomarkers such as procalcitonin and pre-sepsin. A longitudinal study may also assess the benefit of AEC in the prediction of long-term outcomes in patients with CLD and out of hospital mortality as well as other clinical outcomes.
